# The association of rainfall and Buruli ulcer in southeastern Australia

**DOI:** 10.1371/journal.pntd.0006757

**Published:** 2018-09-17

**Authors:** Arvind Yerramilli, Ee Laine Tay, Andrew J. Stewardson, Janet Fyfe, Daniel P. O’Brien, Paul D. R. Johnson

**Affiliations:** 1 University of Melbourne, Department of Medicine, Austin Health, Heidelberg, Victoria, Australia; 2 Department of Medicine and Infectious Diseases, The Royal Melbourne Hospital, University of Melbourne, Parkville, Victoria, Australia; 3 Health Protection Branch, Department of Health & Human Services, Melbourne, Victoria, Australia; 4 Infectious Diseases Department, Austin Health, Heidelberg, Victoria, Australia; 5 Department of Infectious Diseases, Alfred Hospital and Central Clinical School, Monash University, Melbourne, Victoria, Australia; 6 Victorian Infectious Diseases References Laboratory, Peter Doherty Institute for Infection and Immunity, Parkville, Victoria, Australia; 7 Department of Infectious Diseases, Barwon Health, Geelong, Victoria, Australia; Swiss Tropical and Public Health Institute, SWITZERLAND

## Abstract

**Background:**

Buruli ulcer has been increasing in incidence in southeastern Australia with unclear transmission mechanisms. We aimed to investigate the link between rainfall and case numbers in two endemic areas of the state of Victoria; the Bellarine and Mornington Peninsulas.

**Methodology:**

We created yearly and monthly graphs comparing rainfall with local Buruli ulcer incidence for the period 2004–2016 by endemic region and then considered a range of time lag intervals of 0–24 months to investigate patterns of correlation.

**Conclusions:**

Optimal positive correlation for the Bellarine Peninsula occurred with a 12-month prior rainfall lag, however, no significant correlation was observed on the Mornington Peninsula for any time lag. These results provide an update in evidence to further explore transmission mechanisms which may differ between these geographically proximate endemic regions.

## Author summary

Buruli ulcer, a mycobacterial infection resulting in destructive soft tissue lesions, has been increasing in incidence in southeastern Australia over recent years. Exact transmission mechanisms and therefore preventative measures remain unclear. We aimed to investigate a possible link between Buruli ulcer and rainfall for two major endemic areas of the state of Victoria; the Bellarine and Mornington Peninsulas. Our results demonstrate a positive correlation with rainfall on the Bellarine Peninsula with a prior rainfall lag of 12 months but no correlation on the Mornington Peninsula. Established time-frames such as the incubation period and average delay to diagnosis (due to slow and often asymptomatic progression of the disease) make up a total of at least 5–6 months. On the Bellarine Peninsula, there appears to be another 5–6 months of unaccounted time from rainfall to transmission of the pathogen. This provides a basis to explore previously proposed transmission mechanisms which, based on the discrepancy noted with rainfall correlation, may differ between endemic regions.

## Introduction

Buruli ulcer (BU), endemic to west and sub-Saharan Africa, Australia and several other countries, is a potentially severe infection of subcutaneous tissue, caused by the environmental pathogen *Mycobacterium ulcerans* (MU) [1]. Exact transmission mechanisms remain unclear despite new understanding of complex ecological interactions [2, 3]. Increased disease prevalence has previously been linked to areas of forested land cover with low level terrain and proximity to wetlands and swamps [2–5]. Optimal mean temperatures with reduced sunlight and ample oxygen may also play a pivotal role given the pathogen grows best in these environmental conditions [2, 3]. Rainfall has also been shown to be a central driving environmental trigger with recent field studies in Africa linking rainfall with BU diagnoses and an increased detection of MU in the environment [6, 7].

Established time lags between rain and subsequent recognition of incident BU cases may provide a conceptual framework for understanding the ecology and transmission of MU. The first is a long mean incubation period of 4–5 months [8, 9]. Secondly, BU tends to have a slow clinical progression resulting in diagnosis 1–2 months after a lesion is first observed [10, 11]. There is therefore an expected lag of at least 5–6 months between potential permissive environmental events such as increased rainfall and an increase in human disease [8, 9, 11].

Climate is well recognised to influence infectious disease outbreaks through alterations in pathogen, reservoir and vector dynamics as well as influencing human behavior [12, 13]. In southeastern Australia, native mammals such as ringtail (*Pseudocheirus peregrinus)* and brushtail (*Trichosurus vulpecula*) possums have been implicated as reservoir/amplifiers of MU while certain species of mosquitoes have been suggested to act as mechanical vectors [14–18]. Rain may influence the number of possums infected through increased environmental pathogen abundance, subsequent digestion and excretion of contaminated vegetation, and caecotrophic behavior [5, 16]. Further, local salt marsh mosquito population increases are linked to rainfall, with previous studies noting an increase in local abundance after periods of heavy rain [19, 20].

Additional time therefore needs to be considered from an environmental trigger such as rain and the resultant growth of *M*. *ulcerans* in the environment, along with time delays related to potential modes of transmission such as uptake in reservoirs with or without spread by insect vectors. To our knowledge, there have been no studies to date in southeastern Australia which have investigated the longitudinal temporal and seasonal relationship of rain and BU. We therefore aimed to determine whether a correlation exists between these two entities over a 13-year study period including the effect of a range of time lag adjustments to further explore local transmission mechanisms.

## Methods

**Fig 1:**
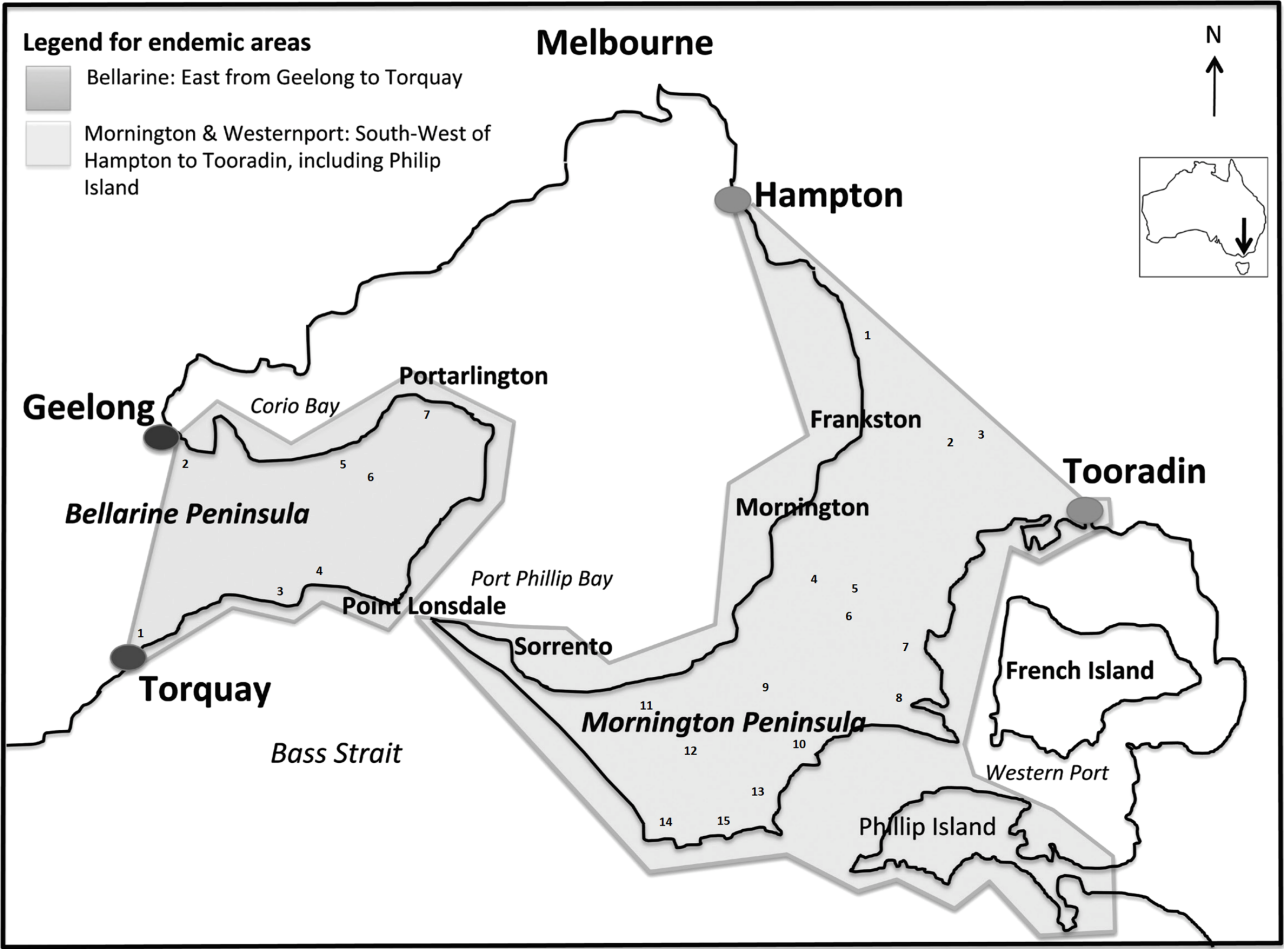
Map of Buruli endemic zones in the southeastern Australian state of Victoria with approximate location of weather stations (numbered). Corresponding numbers for weather stations can be found in Table 3. Figure with adapted text from Trubiano et al [8] as per the Creative Commons Attribution License [https://doi.org/10.1371/journal.pntd.0002463]. Approximate location of weather stations was sourced from the Bureau of Meteorology’s Climate Data Online services [22].

This study is a retrospective analysis of BU notifications data from 2004 to 2016 obtained from the Victorian Government’s Department of Health & Human Services (DHHS) and the Victorian Infectious Diseases Reference Laboratory (VIDRL) [21]. Buruli ulcer was made legally notifiable in the state of Victoria from January 2004 under the Public Health and Wellbeing Act 2008. Aggregated anonymized data used were BU cases by month and year of notification, postcode of residence at the time of notification and the most likely exposure location based on patient history (residence or travel to an endemic area or perceived acquisition in a specific geographical region). In Victoria, BU is acquired in well-defined endemic zones; particularly the Bellarine and Mornington Peninsulas to the southwest and southeast of the city of Melbourne, respectively [8, 9]. We obtained rainfall data from all weather stations in these major endemic areas from the Australian Bureau of Meteorology’s (BOM) Climate Data Online (Fig 1) [22]. Due to very low case numbers since 2004 we excluded two areas from subsequent analyses; Phillip Island, near the endemic region of the Mornington Peninsula, and East Gippsland, located approximately 250km east of Melbourne.

We compared annual monthly rainfall averages with BU incidence per 100,000 person-years in the Bellarine and Mornington Peninsulas from 2004–2016 to examine secular trends. This was achieved by first computing the sum of yearly rainfall across all weather stations with monthly data points (data point = amount of rainfall for a given month in a given year) in an endemic region. The result for each year was then divided by the number of data points available to negate the effect of any missing data points. Finally, these results were divided by the number of weather stations with valid data for those years to produce annual monthly rainfall averages from 2004–2016. BU incidence was defined as the number of BU cases residing in an endemic area divided by the approximate population of the endemic area (defined through postcode region with at least one case of BU over the study period–Table 1) multiplied by 100,000. Population data was sourced from The Australian Bureau of Statistics (ABS) Census data 2005, 2011 and 2016 [23]. To investigate seasonal trends in the two endemic regions, we compared monthly rainfall (averaged over weather stations with valid data points) with the total number of BU cases (both residents and travelers) for each calendar month over the same 13-year period.

**Table 1:** Postcode regions as defined for Buruli ulcer cases from residents in corresponding endemic regions.

Bellarine Peninsula		Mornington Peninsula	
Postcode	Regions	Postcode	Regions
3222	Clifton Springs, Curlewis, Drysdale	3912	Somerville
3223	St Leonards, Indented Heads	3915	Hastings
3224	Leopold	3918	Bittern, Crib Point
3225	Point Lonsdale, Queenscliff	3930	Mt Eliza
3226	Ocean Grove	3931	Mornington
3227	Barwon Heads, Connewarre	3933	Moorooduc
		3934	Mt Martha
		3936	Dromana
		3939	Rosebud, Boneo
		3940	Rosebud West
		3941	Rye, St Andrews Beach, Tootgarook
		3942	Blairgowrie
		3943	Sorrento
		3944	Portsea

Transmission delays were examined by comparing BU incidence with averaged total rainfall (over corresponding weather stations) for the 12-months prior for each of 0 to 24-month time lags which we investigated for each year in the reporting period. Total 12-month prior rainfall was used to match the temporal resolution of incidence calculations which were also derived from 12-month (yearly) BU case totals as defined above. For example, for a 1-month lag, total cases for 2004 would be matched with averaged (across weather stations) 12-month prior rainfall from December 2003 to November 2004 while for a 2-month lag, BU incidence for 2004 would be matched with averaged rainfall from November 2003 to October 2004 and so on for every year from 2004–2016. Annual monthly rainfall averages for each monthly time lag (0–24 months) were then each plotted against BU incidence from 2004–2016, making a total of 25 separate plots. The correlation coefficient for each plot was then calculated and plotted against the degree of time lag (each of 0–24 months) for each endemic region to observe which time lag produced the greatest correlation (S1 Fig).

Statistical analyses were performed with measured outcomes including Pearson correlation coefficients (r) and 95% confidence intervals (CI). The strength of the correlation was defined based on previously described cut-offs by Evans for the absolute value of r: 0.00–0.19: “very weak”; 0.20–0.39: “weak”; 0.40–0.59: “moderate”; 0.60–0.79: “strong” and 0.80–1.0: “very strong” [24]. All graphical and statistical analyses were performed using Graphpad Prism (Prism 7 for Windows, Version 7.01, California, USA). Qualitative inspection from map overlays of terrain, amount of sunlight exposure, as well as minimum and maximum temperatures were performed to determine differences between endemic regions. These map overlays were sourced from BOM Climate Data Online [22]. As the data used in this study were collected under the legislative authority of the Public Health and Wellbeing Act 2008, approval from a Human Research Ethics Committee was not required. Approval for data access for this project was given by DHHS.

## Results

The total number of BU cases notified in Victoria from 2004–2016 was 839 (median = 61, IQR = 32–80) with an overall increase observed during the study period (Table 2). The Bellarine Peninsula, where BU was unknown prior to 1998, had 403 diagnosed cases in the study period (residents + travelers) and 7 weather stations with publicly available records. Out of the 403 diagnosed cases, 253 cases were from residents as determined by postcode region (Tables 1–3). On the Mornington Peninsula, there were 257 cases (residents + travelers) during the study period and 15 weather stations (Tables 1–3). Out of the 257 diagnosed cases, 155 were residents (Table 2). BU was first documented on the Mornington Peninsula in 1990, however after 1998 there were few cases linked to this area until 2012, from when there has been a marked year-on-year increase in notified cases. The remaining 179 cases were located outside the endemic zones, largely distributed sporadically in various other areas of Victoria or, for a small subset, for which precise exposure locations were unknown.

**Table 2:** Total number of Buruli ulcer cases and incidence by year with annual monthly rainfall averages calculated for endemic regions and state-wide for Victoria (no prior rainfall lag).

Year	BU* cases (incidence), Victoria	BU cases (incidence), BP^†^ residents	BU cases (incidence), MP^‡^ residents	Annual monthly rainfall average, BP (mm)	Annual monthly rainfall average, MP (mm)
2004	26 (0.5)	8 (18.2)	1 (0.8)	50.5	69.2
2005	41 (0.8)	19 (43.2)	2 (1.7)	50.4	67.4
2006	61 (1.2)	31 (70.5)	1 (0.8)	27.3	42.1
2007	17 (0.3)	3 (6.8)	1 (0.8)	50.0	59.5
2008	34 (0.6)	18 (41)	0 (0.0)	37.5	53.1
2009	28 (0.5)	13 (26.3)	1 (0.8)	41.4	56.6
2010	32 (0.6)	15 (30.3)	0 (0.0)	61.3	80.9
2011	80 (1.4)	41 (82.9)	5 (3.9)	63.9	77.4
2012	77 (1.4)	34 (68.8)	4 (3.1)	56.1	78.6
2013	65 (1.1)	16 (32.4)	14 (11.0)	45.2	71.8
2014	89 (1.5)	26 (43.6)	17 (12.4)	38.9	57.0
2015	107 (1.8)	17 (28.5)	45 (32.7)	35.6	51.3
2016	182 (2.9)	12 (20.1)	64 (46.6)	50.2	67.2

* Buruli ulcer

† Bellarine Peninsula

‡ Mornington Peninsula

**Table 3:** Weather stations with recorded rainfall data from the Mornington and Bellarine Peninsulas. Consecutive missing data for 1 year or more noted below. Number ID corresponds to locations of weather stations in Fig 1.

Number ID	Bellarine Peninsula	Mornington Peninsula
1	Torquay Golf Club^#^	Bonbeach (Carrum)
2	Breakwater (Geelong Racecourse) ^¶^	Cranbourne South
3	Barwon Heads Golf Club	Cranbourne Botanical Gardens
4	Ocean Grove	Mornington
5	Clifton Springs^§^	Mooroduc*
6	Drysdale (Brimdale)	Devilbend^†^
7	Portarlington	Hastings
8		Cerberus
9		Dromana Sussex Farm
10		Merricks Stonier's Winery
11		Rosebud (Country Club)
12		Main Ridge^‡^
13		Shoreham
14		Cape Schanck^¥^
15		Flinders

# No records after 2010

¶ No records prior to 2011

§ No records prior to 2004

* No records after 2013

† No records after 2011

‡ No records prior to 2008

¥ No records for 2014 and 2015

**Fig 2:**
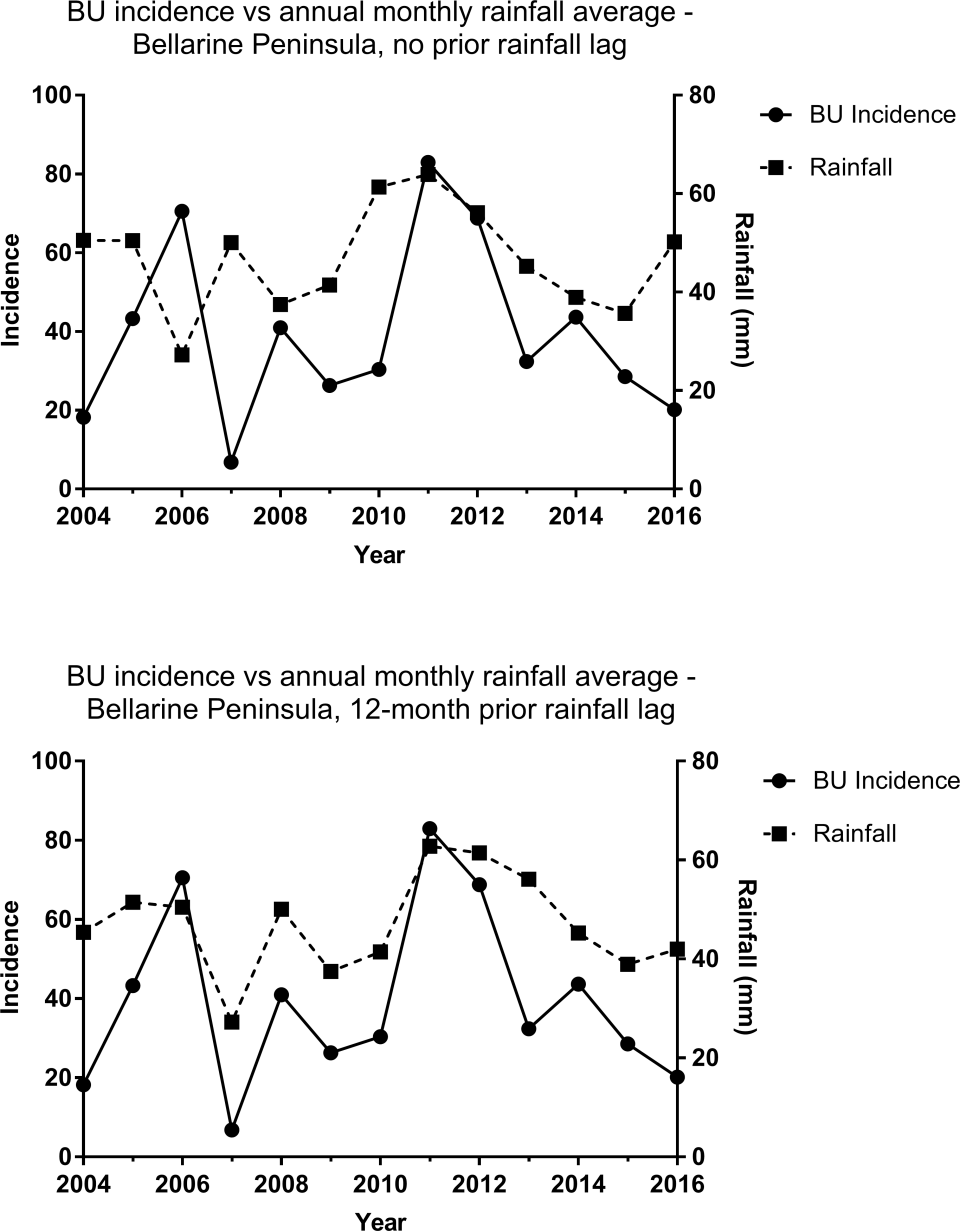
Yearly BU incidence compared to annual monthly rainfall averages for the Bellarine Peninsula from 2004–2016. Fig 2A (top) shows comparisons with no prior rainfall lag (0-month lag) while Fig 2B (bottom) shows case totals compared to 12 months prior rainfall lag (12-month lag). BU = Buruli ulcer, BP = Bellarine Peninsula.

**Fig 3:**
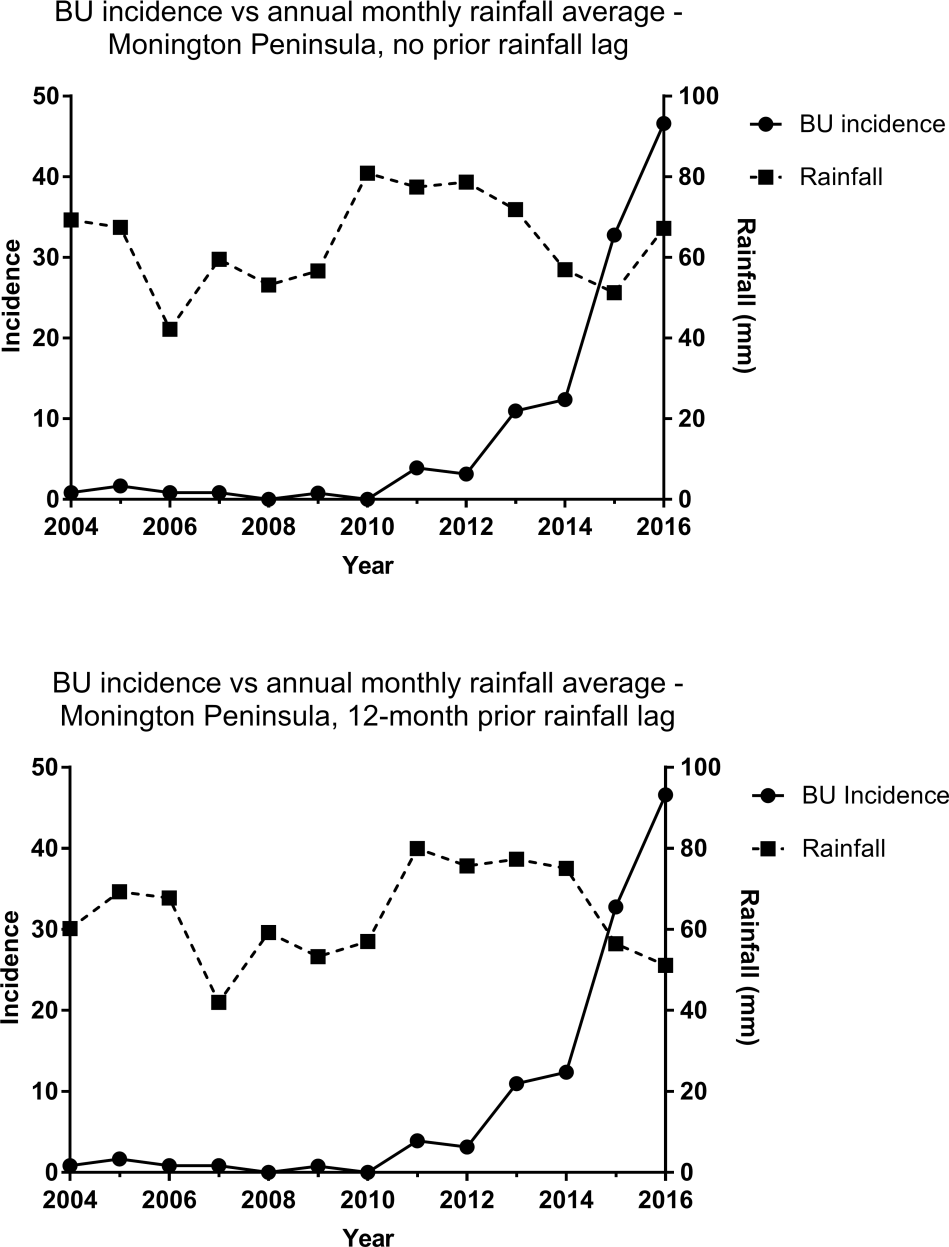
Yearly BU incidence compared to annual monthly rainfall averages for the Mornington Peninsula from 2004–2016. Fig 3A (top) shows comparisons with no prior rainfall lag (0-month lag) while Fig 3B (bottom) shows case totals compared to 12 months prior rainfall lag (12-month lag). BU = Buruli ulcer, MP = Mornington Peninsula.

Comparisons of raw data (0-month lag) from 2004–2016 within each endemic zone revealed very weak correlation between the number of BU diagnoses and annual monthly rainfall averages (Bellarine Peninsula; r = 0.07, 95%, CI = -0.50–0.60, Mornington Peninsula; r = -0.09, 95% CI = -0.61–0.48). Adjusting for total 12-month prior rainfall lags from 0–24 months, optimal with very strong positive correlation for the Bellarine Peninsula occurred with a 12-month lag (r = 0.82, 95% CI = 0.49–0.94) (Fig 2). No moderate to very strong correlations were observed for the Mornington Peninsula regardless of the degree of time lag (Fig 3). Additionally, from qualitative inspection of map overlays using BOM Climate Data Online, the amount of sunlight exposure, maximum and minimum temperatures, as well as elevation above sea level did not differ greatly between endemic regions [22].

**Fig 4:**
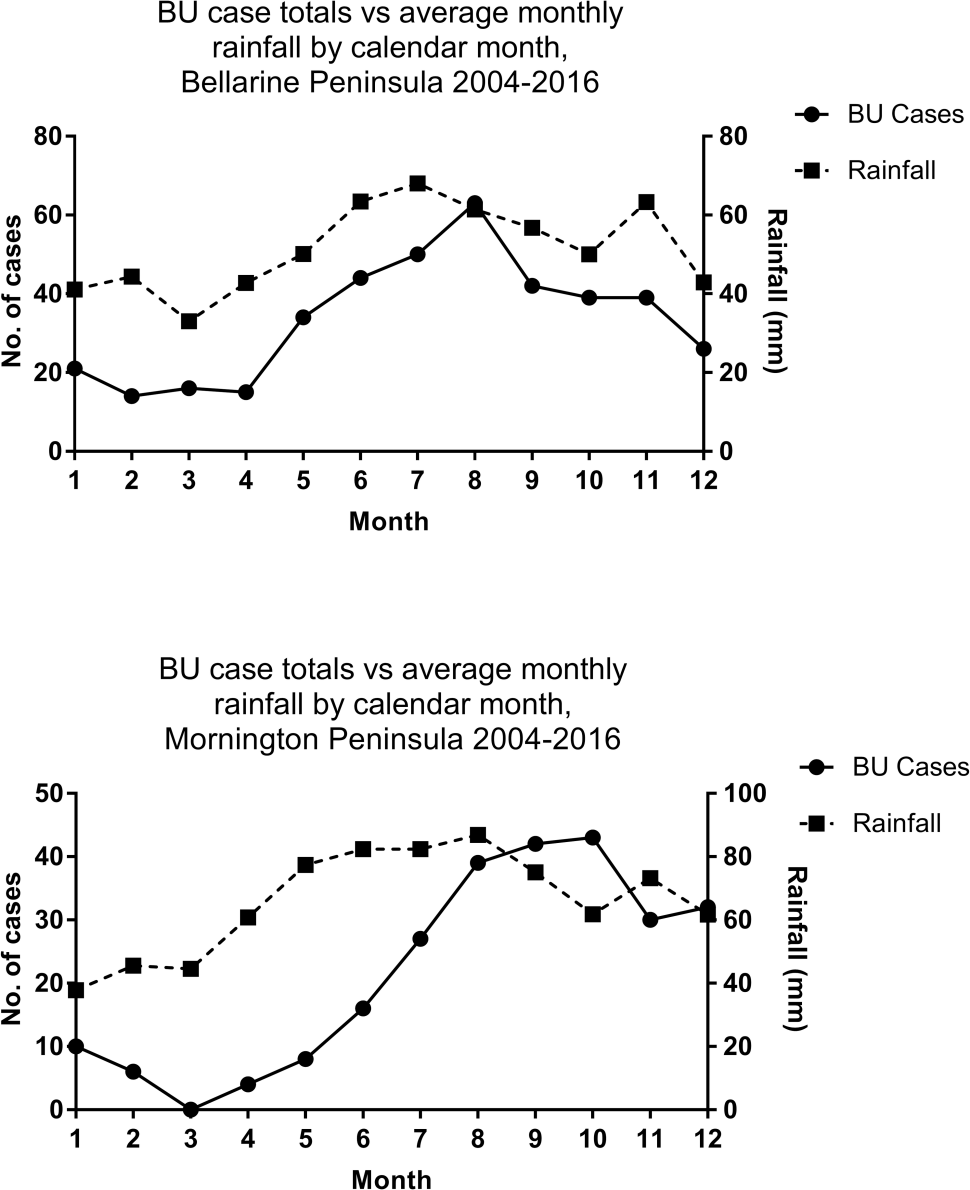
**Total Buruli ulcer cases compared to averaged monthly rainfall, for each calendar month, from 2004–2016 on the Bellarine Peninsula (Fig 4A; top) and Mornington Peninsula (Fig 4B; bottom).** Numbers 1–12 on the x-axes represents months Jan-Dec respectively. BU = Buruli ulcer.

Analysis of seasonal trends revealed most rain falls in the Buruli-endemic regions over the winter months of June, July and August with an isolated spike towards the end of the year in November (Fig 4). Comparison of rainfall between endemic regions resulted in a very strong positive correlation (r = 0.94, 95% CI = 0.79–0.98), suggesting the amount and pattern of rainfall was similar between the two regions over the study period. On the Bellarine Peninsula, high rainfall in the winter months also corresponded with the greatest number of BU diagnoses. Statistical analysis of cases in this region compared to rainfall by calendar month (Fig 4A) revealed very strong positive correlation throughout the calendar year over the 13-year study period (r = 0.86, 95% CI = 0.56–0.96). Cases peaked on the Mornington Peninsula during the spring season months of September and October when rainfall was higher than summer but lower than in winter. Compared to the Bellarine Peninsula, only a moderate positive correlation was noted in this region (Fig 4B) when comparing cases with rainfall throughout the calendar year (r = 0.53, 95% CI = -0.06–0.84).

## Discussion

We have shown a clear correlation between rainfall and BU incidence in one endemic region of Victoria, the Bellarine Peninsula, over a 13-year period when we applied a 12-month prior rainfall lag. The degree of time lag is supported by seasonal trends with rainfall very strongly correlating with the number of BU diagnoses on the Bellarine Peninsula throughout a given calendar year. That is, the amount of rainfall at any period in time in a calendar year strongly correlates with the number of cases of BU at the same point in time or by intervals of one whole calendar year. Accounting for recognised time lags of 5–6 months from transmission of MU to clinical diagnosis, there appears to be an additional time lag of 5–6 months after rain events in this region. We also provide evidence for a recent exponential rise of BU incidence in the other major endemic area of the Mornington Peninsula from 2012. However, no correlation with rainfall was observed on the Mornington Peninsula regardless of the degree of time lag we applied. This result may therefore suggest differences in MU ecology and transmission between endemic zones despite their geographical proximity.

At present, we have no clear explanation for the discrepancy in rainfall correlation between these two major endemic regions of Victoria. However, there is increasing evidence that mosquitoes may act as mechanical vectors in Victoria, acquiring *M*. *ulcerans* directly or indirectly from infected possums which shed MU in their faeces [14–18]. On the Bellarine peninsula, salt marsh mosquitoes (*Aedes camptorhynchus*) are the major pest species and their numbers increase rapidly after significant rain events or high tides and warm weather [14, 19, 20]. As a salt marsh breeding species, adult *A*. *camptorhynchus* generally lay their eggs in damp soil. After rainfall occurs there is a subsequent rise in soil water levels in turn resulting in a drop in dissolved oxygen which stimulates hatching [19, 25]. An expected lag from rainfall is thus favourable to allow for these ecological events with a 1-month lag previously documented by Barton et al as producing the greatest species abundance in the East Gippsland area [19]. This research group also found that mosquito numbers peak in November with a steady reduction every month thereafter through to April. Due to potential differences in landscape and other climate factors, however, the seasonal variability and correlation with rainfall of *A*. *camptorhynchus* may differ on the Bellarine Peninsula, where *M*. *ulcerans* DNA was found in these species, compared to East Gippsland.

Nevertheless, this 1-month lag with rainfall and increased species abundance may fit with our 12-month rainfall-to-diagnosis model. With rainfall peaking in August, there may be an increased abundance of mosquitoes shortly after causing an increase in infection among possum reservoirs such as the common ringtail. Furthermore, rainfall has been shown to significantly increase the survival of the common ringtail possum compared to periods of drought [26]. Thus, an increase in infected possums and contaminated mosquito populations would further contribute to environmental contamination and the overall increasing environmental abundance of *M*. *ulcerans*. The subsequent isolated peak in rainfall in November may then lead to another peak in mosquito abundance from late November through to December. It is during this period, the beginning of the hot Australian summer, that human hosts are most vulnerable with an increased tendency to wear less protective clothing, a well-documented risk factor for developing the disease [2]. Furthermore, in a prior study, we confirmed the distribution of Buruli lesions on the human body to be highly specific, localised to areas such as the distal limb surfaces which tend to be more exposed in warmer months [27]. We also confirmed the summer months of Dec-Feb as the most probable date of infection for a large cohort of patients (n = 338) by using diagnosis dates and back calculating from the already established lag times of 5–6 months (incubation period plus delay to diagnosis) [27]. From acquisition of the pathogen in Dec-Feb, adding another 5–6 months would result in diagnosis in the winter months of Jun-Aug which is consistent with our seasonal observations in the present study.

On the more densely populated Mornington peninsula, *Aedes notoscriptus* are likely to predominate [personal communication, Peter Mee: AgriBio/DEDJTR]. Compared to *A*. *camptorhynchus*, *A*. *notoscriptus* is a container breeder and therefore develops in natural or artificial water-holding containers. *M*. *ulcerans* DNA has been detected in both species but the breeding habits of *A*. *notoscriptus* implies less reliance on rainfall and more on constant water inundation such as the watering of gardens and the use of bore water that continue all year round and are likely to increase in hot, dry weather when *A*. *camptorhynchus* die off. Other possibilities including movement of the pathogen into a new reservoir on the Mornington Peninsula that is less dependent on rain such as permanent wetlands supported by irrigation, or an increasing prevalence in possums. Furthermore, population dynamics, altered human behavior including travel predilection, the abundance of natural water bodies and different pathogen environmental niche requirements may also contribute to the discrepancy noted in rainfall correlation between regions.

A major limitation of our study is that rain was the only environmental variable quantified as opposed to others such as humidity, temperature, sunlight exposure and ground elevation which may all also influence the number of BU diagnoses [2]. From qualitative inspection of map overlays using the Bureau of Meteorology Climate Data Online services, average minimum and maximum temperatures, as well as the amount of sunlight exposure and elevation above sea level, do not appear to differ greatly between endemic areas; however, these need to be further explored[20]. Other environmental niche characteristics for MU such as humidity levels, the availability of nutrients and ample oxygen are recognizably difficult to account for. These factors may alter the eco-biological relationship between climate factors such as rain and BU incidence with a linear correlation perhaps being too simplistic. Nevertheless, demonstrating a linear correlation between the two entities reinforces the importance of rainfall in BU disease and must therefore be considered in a model with other eco-biological factors in future investigations.

Additionally, occurrences of missing rainfall data for individual weather stations may have resulted in systematic errors (Table 2). Most missing data points, however, were sporadic and these were excluded from our calculations by averaging over a large set of valid data points. The impact on resultant trend analyses is therefore unlikely to be significant. The incidence calculations may have also led to systematic errors due to a limitation of 5-yearly Census datasets collected by ABS whereas population rates are likely to have fluctuated on a yearly basis. Nevertheless, we excluded travelers who had contracted BU from our study and only used case totals of patients who reside in endemic regions with population data sourced for postcodes within these regions only. For these reasons, discrepancies in incidence are also unlikely to significantly alter our principal findings.

To investigate time lags, a maximum of 24 months with intervals of 1 month was used to allow meaningful comparison between the results of previous studies [5–7]. One such study by van Ravensway et al using Victorian data has shown a period of high rainfall 19 months, along with drier conditions 5 months prior, to correlate with increased BU emergence [5]. However, this study used case data from 1981–2008 when case numbers were relatively sparse compared to the last decade and examined climate variables with case data from specific points in time rather than investigating longitudinal temporal and seasonal relationships. Another study in an endemic area of Cameroon, Africa, proposed a 5–6 month delay from the high rainy season (Aug-Oct) and peaks in BU incidence (Mar-Apr) [6]. In this instance, the discrepancy with our results may be explained by the absence of an intermediate small animal reservoir which has never been confirmed in Africa [2]. Nevertheless, human-body lesion distribution, which remains highly specific, appears to be similar across countries suggesting unifying global transmission mechanisms [10, 27, 28]. Evidence provided by our study will allow for more focused future investigations of BU transmission taking into consideration time frames for potential modes of transmission, in an overall effort to prevent the spread of disease.

## Supporting information

S1 Checklist STROBE checklist. (DOCX)

S1 Fig Correlation plot for monthly time lags. Correlation coefficient compared with time lags for each of 0–24 months of annual monthly rainfall averages vs annual Buruli incidence (2004–2016) on the Bellarine Peninsula (top) and Mornington Peninsula (bottom). BU = Buruli ulcer.
(TIF)
