# Supplementary figures and images for: The association of rainfall and Buruli ulcer in southeastern Australia
Source: PLoS Negl Trop Dis. 2018 Sep 17;12(9):e0006757. doi: 10.1371/journal.pntd.0006757 (PMC6160213; doi:10.1371/journal.pntd.0006757)

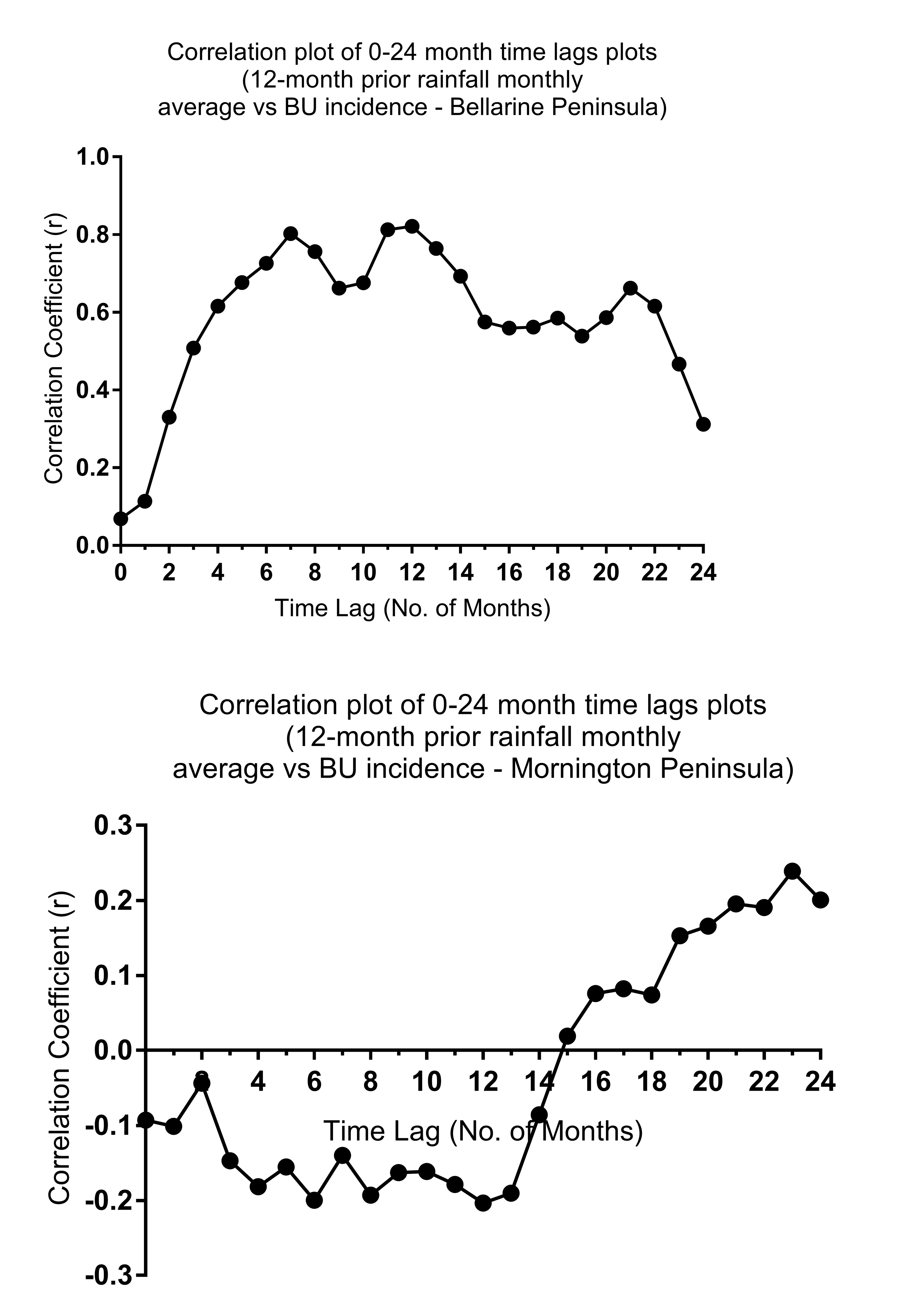

Supplement: S1 Fig — Correlation coefficient compared with time lags for each of 0–24 months of annual monthly rainfall averages vs annual Buruli incidence (2004–2016) on the Bellarine Peninsula (top) and Mornington Peninsula (bottom). BU = Buruli ulcer. (TIF) [file pntd.0006757.s002.tif]
